# Infections à méningocoque lors de purpura fébrile chez l’enfant dans un hôpital marocain: incidence et facteurs cliniques associés

**DOI:** 10.11604/pamj.2017.28.123.6089

**Published:** 2017-10-10

**Authors:** Widad Gueddari, Hayat Sabri, Meryem Chabah

**Affiliations:** 1 Service d’Accueil des Urgences Pédiatriques, Hôpital d’Enfants Abderrahim Harouchi, Centre Hospitalier Universitaire Ibn Rochd, Casablanca, Maroc

**Keywords:** Purpura, méningocoque, fièvre, enfants, infection, Purpura, meningococcal, fever, children, infection

## Abstract

**Introduction:**

Le purpura fébrile (PF) fait craindre une infection à méningocoque, et conduit presque toujours à la réalisation d’un bilan et au traitement par une antibiothérapie à large spectre. Notre objectif était de déterminer l’incidence des infections à méningocoque ainsi que les signes cliniques y associés chez les enfants hospitalisés aux urgences pour purpura fébrile.

**Méthodes:**

Notre étude était descriptive, rétrospective, menée sur une période de 3 ans au service d’accueil des urgences Pédiatriques de l’Hôpital Universitaire d’Enfants de Casablanca. Les enfants inclus étaient ceux hospitalisés pour PF et qui avaient bénéficié d’une hémoculture, associée ou non à une ponction lombaire. Le logiciel SPSS v.16 a été utilisé pour l’analyse statistique.

**Résultats:**

Nous avons inclus 96 enfants dont 49 garçons et 47 filles. La moyenne d’âge était de 53,3 ± 40,5 mois. La moyenne de la température corporelle était de 38,9°C. Une infection à méningocoque a été diagnostiquée chez 35/96 enfants. Une méningococcémie était retenue chez 22 enfants, associée à une méningite chez quatre. Les symptômes et signes physiques significativement associés à une infection à méningocoque étaient la léthargie (p = 0,04), les convulsions (p = 0,01) et la localisation du purpura en dehors du territoire cutané drainé par la veine cave supérieure (p = 0,01).

**Conclusion:**

Un PF localisé en dehors du territoire cutané drainé par la veine cave supérieure ou associé à des convulsions est fortement associé à une infection à méningocoque dont l’incidence semble élevée chez l’enfant marocain.

## Abstract (en)

**Introduction:**

Febrile purpura (FP) is suggestive of meningococcal disease, requiring almost always further investigations and a treatment based on broad spectrum antibiotics. This study aimed to determine the incidence of meningococcal infections as well as their associated clinical signs in children with febrile purpura hospitalized in the emergency department.

**Methods:**

We conducted a descriptive, retrospective study in the pediatric emergency department at the Children’s Hospital of Casablanca over a period of 3 years. The hospitalized children with FP who had undergone bloodculture, whether or not associated with lumbar puncture, were included in the study. Statistical analysis was performed using SPSS v.16 software.

**Results:**

We enrolled 96 children, 49 boys and 47 girls. The average age was 53.3 ± 40.5 months. Mean body temperature was 38.9°C. Meningococcal infection was diagnosed in 35/96 children. The diagnosis of meningococcemia was retained in 22 children, associated with meningitis in four patients. Symptoms and physical signs significantly associated with meningococcal infection included lethargy (p = 0.04), convulsions (p = 0.01) and purpura occurring outside the skin area drained by the superior vena cava (p = 0.01).

**Conclusion:**

FP occurring outside the skin area drained by the superior vena cava or associated with convulsions is srongly related to meningococcal infection, whose incidence seems to be high among Moroccan children.

## Introduction

Le purpura est un motif fréquent de consultation en pédiatrie. Son association avec la fièvre fait craindre une infection. Celle-ci peut être d’origine virale ou bactérienne, mais la gravité de l’infection bactérienne notamment à méningocoque, dont la mortalité peut atteindre 17% [1-3], fait du purpura fébrile une urgence diagnostique et thérapeutique. Au Maroc, la pathologie infectieuse reste une cause importante de mortalité infantile [4]. Le ministère de la santé marocain a généralisé le vaccin contre l’Haemophilus de type B en 2007 et le vaccin contre le Pneumocoque en 2010, afin de diminuer la mortalité liée à ces bactéries invasives. Cependant, le vaccin contre le méningocoque n’est pas inclus dans le programme national d’immunisation. En l’absence de recommandations locales concernant la prise en charge du purpura fébrile (PF), les pratiques professionnelles sont variables. Dans notre Service d’Accueil des Urgences Pédiatriques (SAUP) le médecin de garde hospitalise tout enfant consultant pour PF; il réalise un bilan comportant une hémoculture, une numération globulaire avec formule sanguine, un bilan d’hémostase, un dosage de la C-Réactive Protéine et quand c’est possible une ponction lombaire, et démarre une antibiothérapie à base de céphalosporine de 3^ème^ génération. Dans l’objectif de déterminer l’incidence des infections à méningocoque et le profil épidémiologique des enfants admis pour PF, nous avons réalisé une étude rétrospective transversale.

## Méthodes

Notre étude était descriptive, rétrospective, réalisée sur une période de 3 ans, du 1 janvier 2011 au 31 décembre 2013, au Service d’Accueil des Urgences Pédiatriques (SAUP) de l’Hôpital d’Enfants du Centre Hospitalier Universitaire Ibn Rochd de Casablanca. C’est le seul centre hospitalier spécialisé en urgence pédiatrique pour le grand Casablanca, avec en moyenne 50 000 passages par an et 80 enfants hospitalisés par mois. Les enfants inclus étaient ceux hospitalisés pour purpura fébrile et ayant bénéficié d’un bilan bactériologique fait d’une hémoculture avec ou sans ponction lombaire. La fièvre était définie comme une température rectale supérieure ou égale à 38°C, prise par le personnel médical au SAUP ou par les parents au domicile. Les critères d’exclusion étaient: dossier médical incomplet, prise d’antibiotique avant le prélèvement bactériologique. Les variables de l’étude étaient: les critères démographiques de chaque malade (âge, sexe), les antécédents (contact méningococcique, infection des voies aériennes supérieures, méningite, vaccination contre le méningocoque), les symptômes rapportés par l’enfant ou par un de ses parents, les signes physiques décrits par le médecin ayant hospitalisé l’enfant, et les résultats bactériologiques. Un trouble hémodynamique était retenu devant la présence d’une tachycardie et un temps de recoloration cutanée (TRC) allongé avec ou sans hypotension artérielle. Un état septique était défini par la présence d’une fièvre dépassant 38,5°C associée à une tachycardie ou à une tachypnée. Les taches purpuriques étaient décrites selon leur type (pétéchial, ecchymotique ou nécrotique), et leur localisation (visage et tronc au-dessus de la ligne mamelonaire, membre inférieur, extensif à tout le corps). Un syndrome d’irritation méningée était défini par la présence d’une raideur de la nuque associée ou non à des céphalées et/ou à des vomissements. Une infection à méningocoque était retenue devant l’isolement du Neisseria meningitidis sur l’hémoculture et/ou sur la culture du liquide céphalorachidien (LCR). Le recueil des données a été fait à l’aide d’une fiche d’exploitation à partir des dossiers des malades. L’analyse statistique a été faite à l’aide du logiciel SPSS.v.16. Le seuil p < 5% était considéré comme significatif et l’intervalle de confiance était choisi égal à 95%.

## Résultats

**Figure 1:**
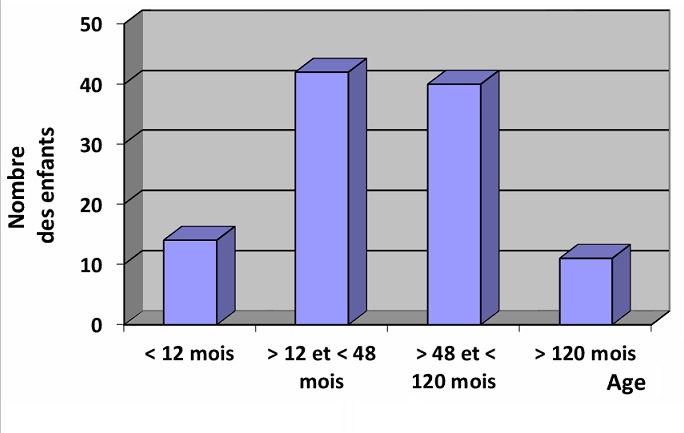
Le nombre des enfants hospitalisés pour purpura fébrile selon l’âge

Nous avons inclus 96 enfants dont 49 garçons et 47 filles. La moyenne d’âge était de 53,3 mois (ET = 40,5). Quatre-vingt-deux enfants étaient âgés de plus de 12 mois, dont 40 de plus de 4 ans (Figure 1). La moyenne de la température corporelle était de 38,9°C avec une minimale de 38°C et une maximale de 41°C. Dans les antécédents des enfants inclus, il y avait une infection des voies aériennes supérieures chez un enfant, un contact méningococcique chez trois enfants et deux enfants avaient une méningite non identifiée. Chez les 96 enfants inclus pour PF, 35 avaient une infection à méningocoque. Une méningococcémie était retenue chez 22 enfants, associée à une méningite chez quatre d’entre eux. D’autres bactéries ont été isolées sur les hémocultures de 4 enfants (Tableau 1). Chez les 88 enfants qui avaient bénéficié d’une ponction lombaire pour suspicion de méningite, 13 avaient une méningite à méningocoque, et 7 avaient une réaction inflammatoire en faveur d’une méningite bactérienne sans germe isolé. Les principaux symptômes étaient l’éruption cutanée rapportée par les parents (74,2%), les vomissements (43,8%), les céphalées (31,3%). Le reste des symptômes figure dans le Tableau 2. La date d’apparition des symptômes variait entre 3h et 7 jours. Tous les malades avaient un purpura, qui était pétéchial dans 53,8% des cas, ecchymotique dans 42% des cas et nécrotique dans 3,3% des cas. Les signes physiques associés étaient des troubles hémodynamiques dans 56,3% des cas, un syndrome d’irritation méningée dans 29,2% des cas, des convulsions chez 4 enfants. Les taches purpuriques étaient extensives à tout le corps dans 71,6% des cas, localisées au niveau des membres inférieurs dans 20% des cas, et au niveau du visage et du tronc au-dessus de la ligne mamelonaire dans 8,4% des cas. Elles évoluaient depuis 1 à 48 heures. La répartition en deux groupes des enfants inclus, selon la présence ou non d’une infection à méningocoque ou à d’autres bactéries, montre que les céphalées et les vomissements étaient les principaux symptômes; et les troubles hémodynamiques étaient présents chez presque les 2/3 d’enfants qui n’avaient aucune bactérie à la culture. Par contre, aucun des enfants infectés par le méningocoque n’avait de taches purpuriques localisées seulement au visage et au tronc au-dessus de la ligne mamelonaire (partie du corps drainée par la veine cave supérieure). Les symptômes et signes physiques qui étaient significativement associés à une infection à méningocoque étaient la léthargie (p = 0,04), les convulsions (p = 0,01) et la localisation du purpura en dehors du territoire cutané drainé par la veine cave supérieure (p = 0,01) (Tableau 3). Dans le groupe d’enfants infectés par le méningocoque, 19 étaient âgés de 12 mois à moins de 5 ans, 13 de 5 ans à 13 ans, et uniquement 3 enfants étaient âgés de moins de 12 mois.

**Tableau 1:** Les bactéries isolées sur les hémocultures des enfants admis pour purpura fébrile

Bactéries isolées sur les hémocultures	Nombre N= 26
Méningocoque	22
Bacillus	2
Acénitobacter	1
Staphylocoque coagulase négative	1

**Tableau 2:** Les principaux symptômes présentés par les enfants hospitalisés pour purpura fébrile

Symptôme	Pourcentage (%)	IC 95%
Eruption cutanée	74,2	[73,38 – 75,38]
Vomissements	43,8	[33,8 – 53,72]
Céphalées	31,3	[22 – 40,5]
Diarrhée	12,5	[5,89 – 19,11]
Douleur de la nuque	10,4	[7,29 – 13,5]
Arthralgie	9,4	[3,57 – 15,23]

**Tableau 3:** Les symptômes et signes physiques chez les enfants infectés par le méningocoque et chez ceux ayant une culture stérile

Symptômes et signes physiques	Infections à méningocoque N= 35	Culture stérile N= 57	P value
Céphalées	11 (31,4%)	19 (33,3%)	1
Vomissements	14 (40%)	28 (49,1%)	0,67
Diarrhée	4(11,4%)	8 (14%)	
Douleur de la nuque	6 (17,1%)	4 (7%)	0,16
Douleur abdominale	2 (5,7%)	4 (7%)	1
Arthralgie	4 (11,4%)	5 (8,7%)	0,72
Eruption cutanée	23 (65,7%)	46 (80,7%)	0,32
Léthargie	17 (48,5%)	17 (29,8%)	**0,04**
Irritabilité	3 (8,5%)	6 (10,5%)	1
Convulsion	4 (11,4%)	0%	**0,01**
Trouble hémodynamique/état septique	19 (54,2%)	35 (61,4%)	0,83
Syndrome d’irritation méningée	14 (40%)	14 (24,5%)	0,10
**Purpura**			
**Type :**			
Pétéchial	18 (51,4%)	32 (56,1%)	0,97
Ecchymotique	16 (45,7%)	22 (38,5%)	
Nécrotique	1 (2,8%)	2 (3,5%)	
**Purpura Localisation :**			
Visage/membre supérieur	0%	8 (14%)	0,01
Membre inférieur	6 (17,1%)	13 (22,8%)	
Tout le corps	29 (82,8%)	39 (68,4%)	

## Discussion

L’association d’un purpura à la fièvre chez un enfant est considérée comme un des critères de gravité devant un enfant fébrile [5]. Dans les recommandations de l’Institut National de la Santé et des Soins d’Excellence (NICE) publiées en 2010 [6], même en l’absence d’autres signes en faveur d’une infection bactérienne, un bilan sanguin comportant hémogramme, C-réactive protéine, hémoculture, bilan d’hémostase, amplification génique par Réaction de Polymérisation en Chaine (PCR), glycémie et gaz du sang doit être réalisé. Ceci est recommandé pour un diagnostic rapide d’infection à méningocoque, puisqu’elle est la principale bactérie occasionnant un purpura fébrile. Cependant, plusieurs virus peuvent être en cause [7-11]. Dans une étude prospective incluant 190 enfants admis pour purpura fébrile, 13 (7%) avaient une infection à méningocoque, 17 avaient une infection à streptococcus pyogène et 28 avaient une infection virale [7]. Dans notre série, 35 enfants (36,4%) avaient une infection à méningocoque. Les autres bactéries isolées étaient considérées comme une souillure. L’incidence élevée d’infection à méningocoque dans notre étude peut être expliquée par le biais de recrutement hospitalier et par la gravité de l’état des malades adressés à notre service. Toutefois, la recherche du méningocoque n’a été faite que sur une seule hémoculture par malade. La recherche du méningocoque par PCR, dont la spécificité et la sensibilité sont de 96% et 93% respectivement [12], n’a été réalisée chez aucun de nos malades. De ce fait, dans les 57 cas où l’hémoculture n’a pas isolé de germe, ni une infection bactérienne invasive ni une infection virale ne peuvent être éliminées, d’autant plus que la recherche des virus n’a pas été faite chez nos malades.

Les symptômes et signes physiques habituellement associés aux infections à méningocoque ne sont pas spécifiques. Ce qui explique que le diagnostic est parfois difficile. Plusieurs auteurs ont montré qu’un enfant en mauvais état général ou ayant un aspect toxique ou septique est très susceptible d’avoir une infection à méningocoque [7, 13]. Dans notre série, près de la moitié (45,7%) des enfants infectés par le méningocoque étaient en bon état général et n’avaient aucun trouble hémodynamique. Alors que, 35/57 (61,4%) des enfants ayant une culture stérile avaient des troubles hémodynamiques ou un état septique clinique. Brogan et coll [14] ont montré, dans une étude incluant 55 enfants, que les facteurs de risque d’une infection bactérienne invasive en cas de PF étaient l’état de choc (temps de recoloration > 2 sec. et/ou hypotension), l’irritabilité, la léthargie, les anomalies du taux des globules blancs totaux (> 15 000 ou < 5000), et l’augmentation de la C- réactive protéine (> 5 mg/l). Par contre, Riordan et coll [15] ont noté que les critères ILL (irritabilité, léthargie, temps de recoloration allongé) avaient des limites à leur utilisation puisque 62/65 des enfants qui avaient ces critères avaient une infection virale. Nos résultats concordent avec ces données, sauf que la recherche des virus n’a pas été faite.

En dehors de ces signes physiques, il semble que la répartition des taches purpuriques a une valeur prédictive étiologique. Wells et coll [16], dans une étude prospective incluant 218 enfants admis pour purpura d’origine infectieuse, ont montré qu’aucun des 24 malades infectés par le méningocoque n’avait un purpura localisé au niveau du visage et du tronc au-dessus de la ligne mamelonaire (la région du corps drainée par la veine cave supérieure). Nos résultats rejoignent ces données et celles de Baker [7] et de Nelson [17]. En plus de la localisation du purpura, Nelson et coll [17] ont montré que l’aspect hémorragique du purpura et son diamètre > 2 mm ont une Valeur Prédictive Positive d’infection à méningocoque de 97%. Dans notre série, le purpura présent chez tous nos malades était essentiellement pétéchial mais extensif à tout le corps dans 71,6% des cas. Cependant, le diamètre des taches purpuriques n’a pas été mesuré chez nos malades.

Concernant l’âge des enfants infectés par le méningocoque, la majorité était âgée de plus de 12 mois. En France, le taux maximum d’incidence des infections à méningocoque est enregistré chez les enfants âgés de moins de 1 an [18]. Cependant, la taille de notre population est petite et ne peut représenter la population pédiatrique au Maroc. Tenant compte des résultats de notre étude et des données de la littérature, la conduite pratique de nos médecins était justifiée. Toutefois, la petite taille de notre population ne nous a pas permis la détermination des facteurs prédictifs d’infection à méningocoque en cas de purpura fébrile. En outre, le caractère rétrospectif de l’étude était à l’origine de plusieurs données manquantes notamment biologiques.

## Conclusion

L’incidence des infections à méningocoque en cas de PF semble être très élevée dans la population d’enfants admis au service des urgences pédiatriques. Ceci justifie la réalisation d’un bilan infectieux et le démarrage d’une antibiothérapie visant le méningocoque devant tout cas de PF. Mais, une étude prospective et multicentrique est indispensable pour une estimation épidémiologique nationale de l’incidence et de la prévalence des infections à méningocoque en cas de PF et pour y déterminer les facteurs prédictifs d’infection bactérienne sévère.

### Etat des connaissances actuelles sur le sujet

- Un PF est très suggestif d’infection à méningocoque si associé à un mauvais état général, un aspect toxique ou un temps de recoloration allongé;
- Un purpura fébrile ecchymotique est en faveur d’une infection à méningocoque.

### Contribution de notre étude à la connaissance

- Les signes prédictifs d’infection à méningocoque en cas de PF étaient: les convulsions, la léthargie et la localisation du purpura en dehors du territoire de drainage de la veine cave supérieure;
- Deux tiers des enfants non infectés par le méningocoque ou autre bactérie avaient des troubles hémodynamiques.

## Conflits d’intérêts

Les auteurs ne déclarent aucun conflit d'intérêts.
